# The real-world effectiveness of sucroferric oxyhydroxide in European hemodialysis patients: a 1-year retrospective database analysis

**DOI:** 10.1186/s12882-020-02188-8

**Published:** 2020-12-07

**Authors:** Rosa Ramos, Charles Chazot, Anibal Ferreira, Attilio Di Benedetto, Konstantin Gurevich, Astrid Feuersenger, Melanie Wolf, Hans-Jürgen Arens, Sebastian Walpen, Stefano Stuard

**Affiliations:** 1 NephroCare Spain, Nephrology, Madrid, Spain; 2 NephroCare France, Fresnes, France; 3 NephroCare Vila Franca de Xira, Nephrology, Vila Franca de Xira, Portugal; 4 NephroCare Italy, Medical Direction, Naples, Italy; 5 Fresenius Medical Care Russia, Saint Petersburg, Russia; 6 Fresenius Medical Care Deutschland GmbH, Bad Homburg, Germany; 7 Vifor Fresenius Medical Care Renal Pharma, Nephrology, Glattbrugg, Switzerland; 8 Fresenius Medical Care, Clinical & Therapeutical Governance, Bad Homburg, Germany

**Keywords:** Chronic kidney disease, End-stage renal disease, Hemodialysis, Hyperphosphatemia, Phosphate binder, Sucroferric oxyhydroxide

## Abstract

**Background:**

The iron-based phosphate binder (PB), sucroferric oxyhydroxide (SFOH), demonstrated its effectiveness for lowering serum phosphate levels, with low daily pill burden, in clinical trials of dialysis patients with hyperphosphatemia. This retrospective database analysis evaluated the real-world effectiveness of SFOH for controlling serum phosphate in European hemodialysis patients.

**Methods:**

De-identified patient data were extracted from a clinical database (EuCliD®) for adult hemodialysis patients from France, Italy, Portugal, Russia and Spain who were newly prescribed SFOH for up to 1 year as part of routine clinical care. Serum phosphate and pill burden were compared between baseline (3-month period before starting SFOH) and four consecutive quarterly periods of SFOH therapy (Q1−Q4; 12 months) in the overall cohort and three subgroups: PB-naïve patients treated with SFOH monotherapy (mSFOH), and PB-pretreated patients who were either switched to SFOH monotherapy (PB → mSFOH), or received SFOH in addition to another PB (PB + SFOH).

**Results:**

1096 hemodialysis patients (mean age: 60.6 years; 65.8% male) were analyzed, including 796, 188 and 53 patients in, respectively, the PB + SFOH, mSFOH, and PB → mSFOH groups. In the overall cohort, serum phosphate decreased significantly from 1.88 mmol/L at baseline to 1.77–1.69 mmol/L during Q1–Q4, and the proportion of patients achieving serum phosphate ≤1.78 mmol/L increased from 41.3% at baseline to 56.2–62.7% during SFOH treatment. Mean PB pill burden decreased from 6.3 pills/day at baseline to 5.0–5.3 pills/day during Q1–Q4. The subgroup analysis found the proportion of patients achieving serum phosphate ≤1.78 mmol/L increased significantly from baseline during SFOH treatment in the PB + SFOH group (from 38.1% up to 60.9% [Q2]) and the mSFOH group (from 49.5% up to 75.2% [Q2]), but there were no significant changes in the PB → mSFOH group. For the PB + SFOH group, serum phosphate reductions were achieved with a similar number of PB pills prescribed at baseline prior to SFOH treatment (6.5 vs 6.2 pills/day at Q4). SFOH daily pill burden was low across all 3 subgroups (2.1–2.8 pills/day).

**Conclusion:**

In this real-world study of European hemodialysis patients, prescription of SFOH as monotherapy to PB-naïve patients, or in addition to existing PB therapy, was associated with significant improvements in serum phosphate control and a low daily pill burden.

## Background

Hyperphosphatemia is a frequent consequence of end-stage renal disease caused by the inability of the kidney to excrete excess phosphate [1]. It is a major contributor to chronic kidney disease-bone and mineral disorder (CKD-MBD), which is associated with vascular and soft tissue calcification, and increased cardiovascular morbidity and mortality [1, 2]. Elevated serum phosphate may be an independent risk factor for vascular calcification [3], cardiovascular events and increased mortality in hemodialysis patients [4, 5].

Restriction of dietary phosphate intake and dialytic phosphate removal treatment are usually insufficient to control serum phosphate levels in advanced CKD; therefore, most dialysis patients require treatment with oral phosphate binders to prevent hyperphosphatemia [6, 7]. However, many phosphate binders are associated with a high daily pill burden (which may account for ~ 50% of oral medications taken by dialysis patients) [8], and adverse effects, particularly gastrointestinal intolerance [6]. These factors may reduce treatment adherence and contribute towards increased serum phosphate levels [7, 9]. Despite the availability of oral phosphate binder therapy, data from COSMOS (Current Management of Secondary hyperparathyroidism – a Multicenter Observational Study) [10] show that approximately 40% of European hemodialysis patients have serum phosphate above the National Kidney Foundation’s Kidney Disease Outcomes Quality Initiative (KDOQI) target range (3.5–5.5 mg/dL [1.13–1.78 mmol/L]) [11].

Sucroferric oxyhydroxide (SFOH, Velphoro® [Vifor Fresenius Medical Care Renal Pharma]) is a chewable, non-calcium, iron-based phosphate binder with a low daily pill burden approved in Europe for the treatment of hyperphosphatemia in CKD patients undergoing dialysis. In a 24-week Phase 3 randomized clinical trial and its 28-week extension study [12, 13], SFOH demonstrated equivalent efficacy to sevelamer carbonate in reducing serum phosphate levels, but had a substantially lower mean ± standard deviation (SD) daily pill burden over the 1-year treatment period (3.3 ± 1.3 vs 8.7 ± 3.6 pills/day, respectively) [13].

Several observational database studies of US dialysis patients have subsequently demonstrated that SFOH provides effective control of serum phosphate, with a relatively low daily pill burden [14–17]. However, published data on the real-world effectiveness of SFOH in European dialysis patients are currently limited to a few smaller country-specific studies. One study evaluated outcomes of Portuguese patients receiving online hemodiafiltration (HDF) who were switched to SFOH from another phosphate binder as part of routine care [18]. After switching to SFOH, patients’ phosphate binder pill burden was reduced by 67% (from 6 to 2 pills/day; *p* < 0.001), and the proportion who achieved target serum phosphate of ≤1.78 mmol/L increased from 33.3% at baseline to 45.0% after 6 months’ treatment. The short-term effect of SFOH on CKD-MBD indices and serum ferritin was evaluated in a cohort study of 262 French hemodialysis patients [19]. Treatment with SFOH reduced mean serum phosphate levels (from 1.99 to 1.83 mmol/L after 2 months; *p* < 0.0001) and significantly increased the proportion of patients achieving target serum phosphate of < 1.5 mmol/L, from 12.1 to 25.7% (*p* < 0.0001). Increases in serum ferritin were also observed during SFOH therapy, consistent with the Phase 3 study findings.

The clinical management of hyperphosphatemia in dialysis patients differs between Europe and the US. In Europe, there is more frequent use of HDF [20], the duration of dialysis sessions tends to be longer [21] and the average phosphate binder pill burden is lower [9]. Hence, data relating to the effectiveness of SFOH obtained from observational studies of US hemodialysis patients may not be applicable to European hemodialysis patients.

This retrospective analysis utilized patient data extracted from the European Clinical Database (EuCliD®) [22] to evaluate the real-world effectiveness of SFOH for the control of serum phosphate levels in a large cohort of hemodialysis patients from five European countries: France, Italy, Portugal, Russia and Spain. The impact of SFOH therapy on other CKD-MBD indices, iron-related parameters and concomitant anti-anemic medication use was also assessed.

## Methods

### Patient population and EuCliD® database

The present study analyzed data for adult (≥18 years) hemodialysis patients who were newly prescribed SFOH as part of routine care between January 2015 and January 2019 and received up to 12 months of SFOH treatment. All prescriptions of SFOH and other phosphate binders were made at the discretion of the treating physician as per routine clinical practice.

The EuCliD® database, maintained by Fresenius Medical Care, was initiated in 1999 [22] to collect demographic, clinical, laboratory and prognostic measurements for patients undergoing hemodialysis across a wide network of European dialysis centers. The present analysis was performed using de-identified (pseudonymized) patient data extracted from EuCliD® electronic records of hemodialysis patients from dialysis centers in France, Italy, Portugal, Russia and Spain. All patients provided written informed consent permitting the use of their data for clinical research purposes.

### Data collection, assessments and outcomes

The treatment periods for data assessment were defined as baseline (the 3-month period prior to SFOH prescription) and SFOH follow-up (defined as Q1 to Q4; 12 consecutive months of SFOH therapy). Comparisons were performed quarterly using the baseline quarter as the reference. Patients in Q1 had at least 60 days of SFOH prescriptions recorded. Patients in Q2 were required to have been included in Q1 and to have received at least 60 days of SFOH prescriptions in Q2. The same inclusion criteria were applied to patients included in Q3 and Q4. Patients not included in one treatment period were excluded from subsequent periods. For the comparisons between baseline and Q4, only patients who received SFOH prescriptions for 12 months (with at least 60 days of recorded prescription during each quarter) were included. Hence, these comparisons display the changes in clinical and laboratory parameters that occurred after 12 months of SFOH therapy vs baseline.

Demographics and clinical characteristics at baseline were summarized for the overall patient cohort and by country. The mean number of prescribed phosphate binder pills was recorded at baseline and during SFOH follow-up. Laboratory parameters evaluated at baseline and during SFOH follow-up comprised: CKD-MBD parameters sampled mid-week (serum phosphate, parathyroid hormone [PTH], calcium), hemoglobin and iron parameters (ferritin and transferrin saturation [TSAT]). Anti-anemic therapy use and dose (intravenous [IV] iron and erythropoiesis-stimulating agents [ESA]) and therapeutic vitamin D and calcimimetic use and dose (active vitamin D and cinacalcet) were also recorded. Measurement of all laboratory parameters was performed according to the clinical routine. All measurements taken during baseline and each quarter of the SFOH follow-up period were averaged for each patient per treatment period. Changes in laboratory parameters were evaluated by comparing baseline and SFOH follow-up data (Q1−Q4).

In this analysis, two approaches were used to analyze changes from baseline in serum phosphate during the SFOH follow-up period. First, changes in serum phosphate control during the follow-up period were classified according to the proportion of patients achieving serum phosphate ≤1.78 mmol/L, based on the targets initially defined by the K/DOQI guidelines [11]. Second, the mean serum phosphate measurements per period were calculated for each patient. The Kidney Disease: Improving Global Outcomes (KDIGO) clinical practice guidelines provide different recommendations regarding target serum phosphate levels, suggesting that, in patients with Stage 5 CKD and undergoing dialysis, elevated phosphate levels should be lowered toward the normal range [23, 24]. Hence, data for the proportion of patients who achieved serum phosphate ≤1.45 mmol/L (≤4.5 mg/dL) were also analyzed.

To evaluate the effects of SFOH in different patient populations, serum phosphate and phosphate binder pill burden data were also analyzed separately in three patient subgroups: phosphate binder-naïve patients treated with SFOH monotherapy (‘mSFOH’); phosphate binder-pretreated patients switched to SFOH monotherapy (‘PB **→** mSFOH’); and phosphate binder-pretreated patients who used another phosphate binder in addition to SFOH (in ≥1 follow-up quarter) (‘PB + SFOH’). Patients were defined as ‘phosphate binder-pretreated’ or ‘phosphate binder-naïve’ according to whether they received phosphate binder therapy or not during the 3-month baseline period.

### Statistical analysis

All statistical analyses were conducted using SAS software (SAS Institute Inc. USA), version 9.4 or later. All analyses were exploratory in nature. Data for all eligible patients in the EuCliD® database were included in the analyses to obtain an accurate picture of real-world SFOH use. The following descriptive methods were performed: categorical variables were summarized by frequency and percentage (n, %) of patients by treatment period; continuous variables were summarized by presenting means (± SD) for each treatment period; and subgroup analyses examining changes in serum phosphate were performed for each country. All changes were calculated using baseline as the reference (100%). Changes were calculated based on ‘patient level’ data (i.e., the differences between baseline and the respective quarter were calculated for each patient). For each patient, their baseline value was compared with their respective quarter value. Only patients with one value for baseline and one value for the respective quarter were included in this calculation. These values were subsequently used to calculate descriptive measures of changes. To test for any differences in parameters between baseline and SFOH follow-up, McNemar’s test for dichotomous variables and a paired t test for continuous variables were applied to display exploratory two-tailed *p*-values that, according to the setting of the performed analyses, were unadjusted. *P*-values ≤0.05 were considered statistically significant.

## Results

### Patient disposition and baseline characteristics

**Fig. 1:**
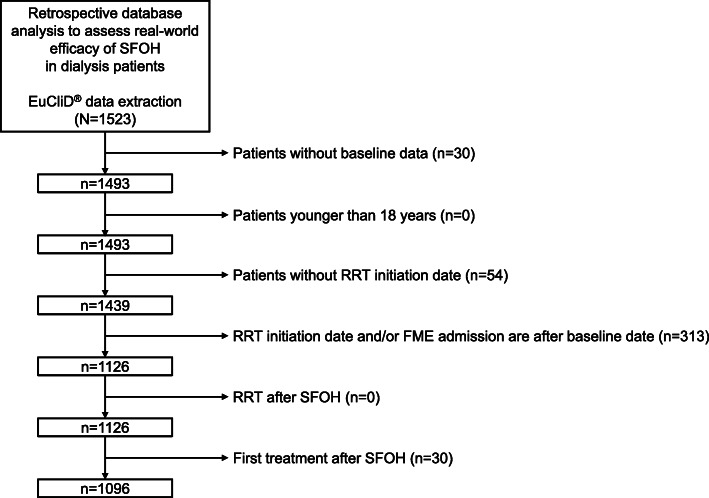
Patient disposition. Abbreviations: EuCliD®, European Clinical Database; FME, Fresenius Medical Care; RRT, renal replacement therapy; SFOH, sucroferric oxyhydroxide

**Fig. 2:**
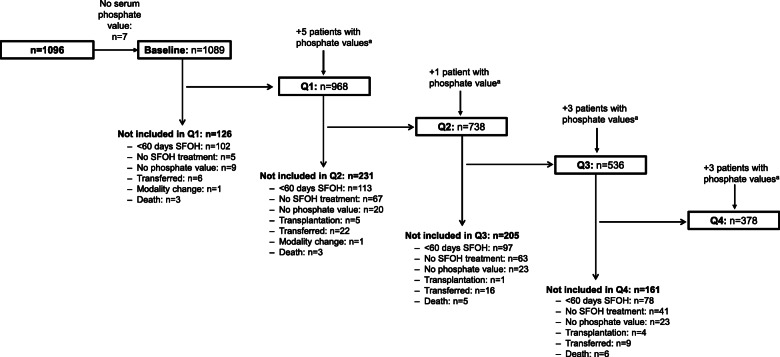
Number of patients analyzed at each assessment period and reasons for exclusion. ^a^Patients with missing serum phosphate values in the previous quarter who could not be analyzed despite receiving SFOH therapy

From a total of 1523 patients with recorded SFOH prescriptions that were captured in the EuCLiD® database, 1096 patients were eligible for inclusion in the final analysis (Fig. 1). Baseline characteristics for the overall study cohort are displayed in Table 1. The majority of patients were male (65.8%) and over half (54.4%) were from Spain. Most patients (62.8%) were receiving HDF, whereas 36.5% were receiving hemodialysis. In total, 968, 738, 536 and 378 hemodialysis patients were eligible for analysis in Q1, Q2, Q3 and Q4, respectively (Fig. 2). The most frequent reason patients were excluded from the analysis at each treatment period was that they had received < 60 days of SFOH treatment. Most patients (78.0%) had received a prior phosphate binder therapy before being prescribed SFOH; the most commonly prescribed regimens included monotherapy with calcium-based phosphate binders (30.3%) or sevelamer (26.2%), and the combination of calcium and sevelamer (22.8%) (Table 1).

**Table 1:** Baseline patient demographics and clinical characteristics

Parameter^a^	Overall study cohort (*N* = 1096)
Sex, n (%)	
Male	721 (65.8)
Female	375 (34.2)
Age, years	60.6 ± 14.8
Country, n (%)	
Spain	596 (54.4)
France	174 (15.9)
Portugal	147 (13.4)
Italy	106 (9.7)
Russia	73 (6.7)
Body mass index, kg/m^2^ [*n* = 804]	27.8 ± 6.0
Dialysis vintage, months	61.0 ± 70.0
≥ 1 year on dialysis, n (%)	857 (78.2)
< 1 year on dialysis, n (%)	239 (21.8)
Dialysis modality^b^	
Hemodiafiltration, n (%)	688 (62.8)
Hemodialysis, n (%)	400 (36.5)
Unknown	8 (0.7)
Prior PB use, n (%)	
Pretreated	855 (78.0)
Naïve	241 (22.0)
Regimen received by PB-pretreated patients, n (%)	*n* = 855
Calcium-based	259 (30.3)
Sevelamer	224 (26.2)
Sevelamer + calcium-based	195 (22.8)
Lanthanum	60 (7.0)
Lanthanum + calcium-based	47 (5.5)
Sevelamer + lanthanum	42 (4.9)
Sevelamer + lanthanum + calcium-based	28 (3.3)
Charlson Comorbidity Index at end of baseline period	3.8 ± 1.9
Age-adjusted Charlson Comorbidity Index at end of baseline period	5.5 ± 2.6
Comorbidities, n (%)^c^	
Hypertension	717 (65.4)
Diabetes	319 (29.1)
Congestive heart failure	274 (25.0)
Peripheral vascular disease	256 (23.4)
Cerebrovascular disease	143 (13.1)
Chronic pulmonary disease	122 (11.1)
Malignant tumor	116 (10.6)
Myocardial infarction	102 (9.3)
Liver disease	82 (7.5)
Peptic ulcer disease	57 (5.2)

^a^Continuous variables are presented as mean ± standard deviation unless otherwise specified

^b^HDF includes online HDF, online single-needle HDF and mixed-dilution HDF, and HD includes single and double-needle HD

^c^Only comorbidities reported for > 5% of patients are shown

*Abbreviations*: *HD* hemodialysis, *HDF* hemodiafiltration, *PB* phosphate binder

Baseline characteristics were generally comparable between countries, with some notable differences (Table 2). Patients in Portugal and Russia were younger than those in France, Italy and Spain. The proportion of patients with comorbidities was higher in Russia and Spain, compared with France, Italy and Portugal. Across the countries, mean dialysis vintage ranged from 44.2 months (France) to 72.0 months (Portugal).

**Table 2:** Baseline demographics and clinical characteristics by country

Parameter^a^	France (***n*** = 174)	Italy (***n*** = 106)	Portugal (***n*** = 147)	Russia (***n*** = 73)	Spain (***n*** = 596)	Overall cohort (*N* = 1096)
Sex, n (%)						
Male	106 (60.9)	69 (65.1)	101 (68.7)	39 (53.4)	406 (68.1)	721 (65.8)
Female	68 (39.1)	37 (34.9)	46 (31.3)	34 (46.6)	190 (31.9)	375 (34.2)
Age, years	62.5 ± 15.4	60.5 ± 14.9	55.3 ± 13.3	54.8 ± 14.7	62.1 ± 14.5	60.6 ± 14.8
Body mass index, kg/m^2^	27.6 ± 6.9	27.5 ± 6.1	26.6 ± 6.0	27.3 ± 5.4	28.1 ± 5.9	27.8 ± 6.0
Dialysis vintage, months	44.2 ± 59.5	71.3 ± 74.1	72.0 ± 68.5	57.3 ± 46.7	61.1 ± 74.4	61.0 ± 70.0
Comorbidities, n (%)						
Diabetes	42 (24.1)	26 (24.5)	29 (19.7)	23 (31.5)	199 (33.4)	319 (29.1)
Congestive heart failure	14 (8.1)	20 (18.9)	14 (9.5)	36 (49.3)	190 (31.9)	274 (25.0)

^a^Continuous variables are presented as mean ± standard deviation unless otherwise specified

### Prior treatment status and concomitant phosphate binder use

The majority of patients from the overall cohort (*n* = 796, 73.1%), who were eligible for analysis at baseline, were phosphate binder-pretreated and prescribed SFOH as an add-on therapy to their prior phosphate binder therapy (‘PB + SFOH’). Fifty-three patients (4.9%) were phosphate binder-pretreated and switched to SFOH monotherapy (‘PB **→** mSFOH’), whereas 188 (17.3%) patients were phosphate binder-naïve and prescribed SFOH monotherapy (‘mSFOH’). A small proportion of patients (*n* = 52, 4.8%) were phosphate binder-naïve and prescribed SFOH in combination with another phosphate binder therapy.

### Changes in serum phosphate

**Fig. 3:**
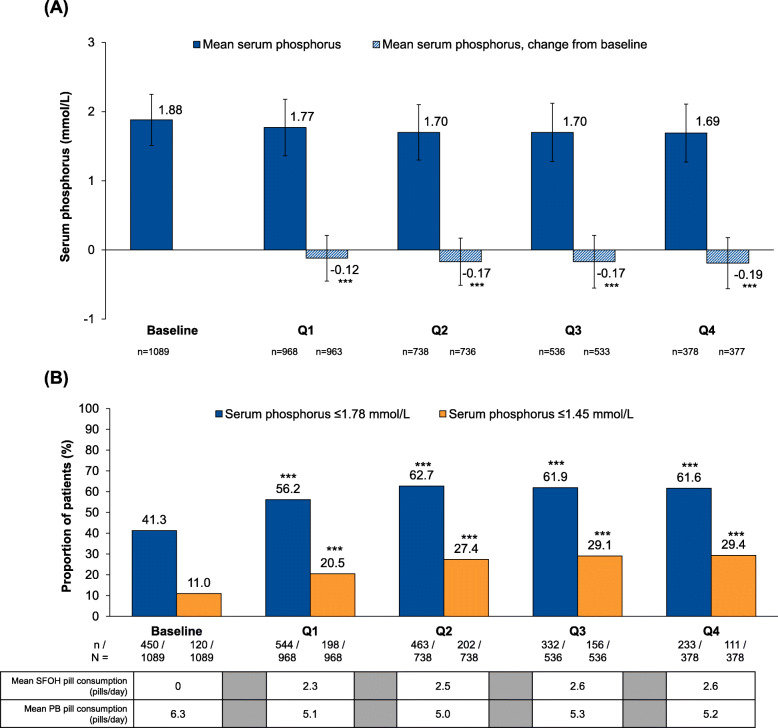
Serum phosphate concentrations during baseline and sucroferric oxyhydroxide follow-up (Q1−Q4). **a** Mean ± SD serum phosphate concentrations. **b** Proportion of patients achieving target serum phosphate of ≤1.78 mmol/L and ≤ 1.45 mmol/L. ***p < 0.0001 (vs baseline). Mean values are shown in the table. Abbreviations: PB, phosphate binder; SD, standard deviation; SFOH, sucroferric oxyhydroxide

**Fig. 4:**
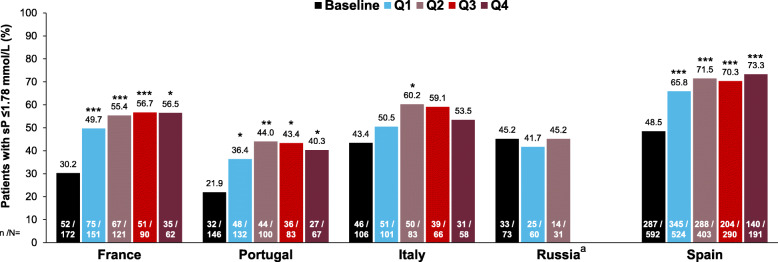
Proportion of patients achieving target serum phosphate (≤ 1.78 mmol/L) by country. *p < 0.05; **p < 0.001; ***p < 0.0001 (vs baseline); ^a^The serum phosphate data for Russia for Q3 and Q4 are not shown because the number of patients with follow-up data available was too low for meaningful analysis (Q3, *n* = 7; Q4, *n* = 0)

In the overall study cohort at baseline, 41.3% of patients had serum phosphate levels below the target (≤1.78 mmol/L), and mean serum phosphorus was 1.88 mmol/L. Following prescription of SFOH, there were significant reductions in serum phosphate levels, from 1.88 mmol/L at baseline to 1.77–1.69 mmol/L during Q1–Q4, respectively (*p* < 0.0001 for each period vs baseline) (Fig. 3a and Table 3). The proportion of patients who achieved serum phosphate ≤1.78 mmol/L increased from 41.3% at baseline to 56.2–62.7% during Q1–Q4 (*p* < 0.0001 for each period vs baseline) (Fig. 3b). Increases from baseline in the proportion of patients achieving serum phosphate ≤1.78 mmol/L were observed in all countries except Russia, possibly due to the relatively low number of patients and the short duration of follow-up (Fig. 4). The proportion of patients from the overall study cohort achieving serum phosphate ≤1.45 mmol/L also increased significantly, from 11.0% at baseline to 20.5–29.4% during Q1–Q4 (*p* < 0.0001, all treatment periods vs baseline) (Fig. 3b).

**Table 3:** CKD-MBD parameters and phosphate binder pill burden at baseline and during sucroferric oxyhydroxide follow-up (Q1 to Q4) in the overall study cohort (*N* = 1096)

Parameter	Baseline (***N*** = 1089)	Q1 (***N*** = 968)	Q2 (***N*** = 738)	Q3 (***N*** = 536)	Q4 (***N*** = 378)
Serum phosphate, mmol/L	1.88 ± 0.36	1.77 ± 0.41^***^	1.70 ± 0.40^***^	1.70 ± 0.42^***^	1.69 ± 0.42^***^
Serum PTH, pmol/L	47.5 ± 46.7	48.8 ± 46.0	48.4 ± 47.2	50.6 ± 52.7	52.1 ± 51.8
Serum calcium, mmol/L	2.24 ± 0.16	2.23 ± 0.17^*^	2.24 ± 0.17	2.24 ± 0.17	2.24 ± 0.16
Total phosphate binder pill consumption, pills/day	6.3 ± 9.0	5.1 ± 7.6	5.0 ± 5.4	5.3 ± 5.6	5.2 ± 5.5
SFOH pill consumption, pills/day	N/A	2.3 ± 1.4	2.5 ± 1.5	2.6 ± 1.5	2.6 ± 1.5
SFOH dose, mg/day	N/A	1172 ± 718	1236 ± 773	1285 ± 770	1308 ± 765
Number of patients receiving cinacalcet, n (%)	355 (32.6)	357 (36.9)	267 (36.2)	200 (37.3)	148 (39.2)
Number of patients receiving vitamin D analogs, n (%)	426 (39.1)	372 (38.4)	293 (39.7)	223 (41.6)	158 (41.8)

*p < 0.05; ***p < 0.0001 (vs baseline)

All values are mean ± SD unless otherwise specified

Abbreviations: *CKD-MBD* chronic kidney disease-bone and mineral disorder, *PTH* parathyroid hormone, *N/A* not applicable, *SD* standard deviation, *SFOH* sucroferric oxyhydroxide

### Phosphate binder pill burden – overall patient cohort

The total phosphate binder and SFOH pill burden (number of pills consumed) at baseline and during SFOH follow-up are summarized in Table 3 and Fig. 3b. The total mean daily phosphate binder pill burden decreased from an average of 6.3 pills/day at baseline (phosphate binder-pretreated patients) to 5.0–5.3 pills/day during Q1–Q4 (overall study cohort).

### Concomitant cinacalcet and vitamin D analog treatment

There was a small increase (from 32.6% at baseline to 39.2% by Q4) in the proportion of patients receiving cinacalcet, whereas the proportion receiving vitamin D analogs remained unchanged during the course of the study (Table 3).

### Serum phosphate changes and pill burden: patient subgroup analysis

**Fig. 5:**
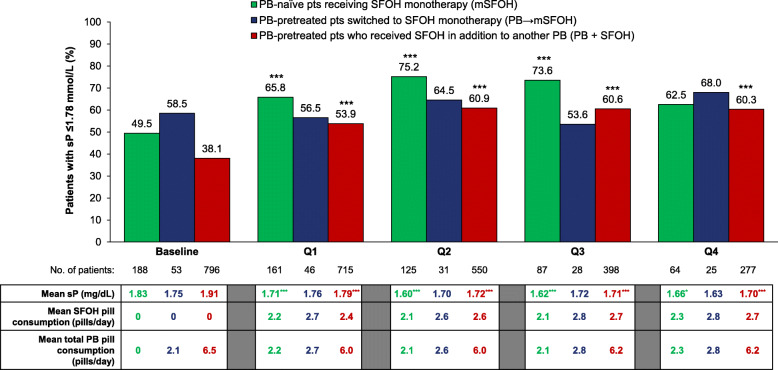
Serum phosphate and phosphate binder pill burden during baseline and sucroferric oxyhydroxide follow-up (Q1–4). Abbreviations: mSFOH, PB-naïve patients treated with SFOH monotherapy; PB, phosphate binder; PB + SFOH, PB-pretreated patients who added SFOH to another PB; PB → SFOH, PB-pretreated patients switched to SFOH monotherapy; pts, patients; SFOH, sucroferric oxyhydroxide; sP, serum phosphate. Mean values are shown in the table. *p < 0.05; ***p < 0.0001 (vs baseline)

A subgroup analysis was performed to evaluate changes from baseline in serum phosphate and pill burden during SFOH therapy in the mSFOH, PB → mSFOH and PB + SFOH patient subgroups (Fig. 5). At baseline, mean serum phosphate levels were highest among PB + SFOH patients (1.91 mmol/L) and lowest among PB → mSFOH patients (1.75 mmol/L). The proportion of patients with serum phosphate ≤1.78 mmol/L at baseline was lowest in the PB + SFOH group (38.1%) and highest in the PB → mSFOH group (58.5%).

During SFOH follow-up, the proportion of patients achieving serum phosphate ≤1.78 mmol/L increased in the mSFOH group (*p* < 0.0001 for Q1, Q2, Q3 vs baseline) and the PB + SFOH group (*p* < 0.0001 for all treatment periods vs baseline) (Fig. 5). In the PB → mSFOH group, there were no statistically significant changes from baseline in the proportion of patients achieving serum phosphate ≤1.78 mmol/L during SFOH follow-up. For PB + SFOH patients, the overall number of phosphate binder pills prescribed at baseline prior to SFOH was similar to the number prescribed during follow-up (6.5 pills/day vs 6.0–6.2 pills/day during Q1–Q4). The total daily pill burden increased slightly for PB → mSFOH patients (2.1 pills/day at baseline vs 2.6–2.8 pills/day during Q1−Q4). The mean SFOH daily pill burden was relatively low across all subgroups (2.1–2.8 pills/day).

### Changes in calcium and parathyroid hormone

A small, but statistically significant reduction in serum calcium from baseline was observed at Q1 (*p* < 0.05 vs baseline), but values remained at baseline levels at the other treatment periods evaluated (Table 3). There was a non-significant trend towards a small increase in plasma PTH during the SFOH follow-up period, from 47.5 pmol/L at baseline to 52.1 pmol/L at Q4.

### Iron-related parameters and anti-anemic medication use

There were small but statistically significant increases in serum ferritin, from 456 μg/L at baseline to 481–502 μg/L during Q1–Q4 (*p* ≤ 0.0026 for all treatment periods vs baseline) (Table 4). There were small increases in TSAT, from 27.9% at baseline to 28.9–29.9% during Q1−Q4 (*p* ≤ 0.003 for Q1 and Q2 vs baseline). Small increases from baseline in hemoglobin levels were also observed during SFOH follow-up, which were statistically significant at Q1 and Q3 (*p ≤* 0.0009 vs baseline).

**Table 4:** Iron-related parameters and concomitant IV iron and ESA use at baseline and during follow-up (Q1 to Q4) in the overall study cohort (*N* = 1096)

Parameter	Baseline (***N*** = 1089)	Q1 (*N* = 968)	Q2 (*N* = 738)	Q3 (*N* = 536)	Q4 (*N* = 378)
Serum ferritin, μg/L	456 ± 331	481 ± 329^**^	502 ± 330^***^	495 ± 320^*^	488 ± 337^*^
Serum TSAT, %	27.9 ± 12.1	29.4 ± 12.0^*^	29.9 ± 12.1^*^	29.3 ± 12.4	28.9 ± 11.6
Hemoglobin, g/L	113.2 ± 12.4	114.7 ± 13.1^**^	113.9 ± 13.9	115.5 ± 12.6^**^	114.9 ± 13.1
Patients receiving IV iron, n (%)	856 (78.6)	739 (76.3)	552 (74.8)^*^	388 (72.4)^*^	279 (73.8)
Mean IV iron dose, mg/week	54.4	51.4	48.5^*^	47.1^*^	50.0
Patients receiving ESA, n (%)	877 (80.5)	751 (77.6)	569 (77.2)	402 (77.1)^*^	282 (74.6)^*^
Mean ESA dose, units/week	6618	5945^***^	5846^*^	6126^*^	5665^*^

*p < 0.05; **p < 0.001; ***p < 0.0001 (vs baseline)

All values are mean ± standard deviation unless otherwise specified

*Abbreviations*: *ESA* erythropoiesis-stimulating agent, *IV* intravenous, *TSAT* transferrin saturation

The percentage of patients administered IV iron therapy decreased slightly from baseline (78.6%) during the SFOH follow-up period (Q1, 76.3%; Q2, 74.8%; Q3, 72.4%; Q4, 73.8%; *p* ≤ 0.0337 for Q2 and Q3 vs baseline) (Table 4). The mean dose of IV iron therapy decreased significantly from 54.4 mg/week at baseline to 48.5 mg/week at Q2 (*p* = 0.0153 vs baseline) and 47.1 mg/week at Q3 (*p* = 0.0137 vs baseline). The proportion of patients receiving ESA decreased progressively during SFOH follow-up, from 80.5% at baseline to 74.6% by Q4 (*p* ≤ 0.0126 at Q3 and Q4 vs baseline), and was accompanied by a significant decrease in the mean dose of ESA therapy from 6618 units/week at baseline to 5665–6126 units/week during Q1–Q4 (*p* ≤ 0.0448 for Q1–Q4 vs baseline).

## Discussion

This retrospective database analysis of > 1000 European hemodialysis patients showed that treatment with SFOH was associated with an improvement in serum phosphate control when prescribed as part of routine practice for up to 1 year. It is noteworthy that the population analyzed in this study comprised a selected group of hemodialysis patients who were prescribed SFOH and therefore it may not be fully representative of the wider EuCliD® patient population. In total, 58.7% of patients in the overall study cohort had serum phosphate levels above K/DOQI target levels (> 5.5 mg/dL [1.78 mmol/L]) at baseline – a slightly higher proportion than the 41% reported for the COSMOS study cohort, which consisted of 4500 European hemodialysis patients [10].

In the present analysis, the proportion of patients in the overall cohort achieving serum phosphate ≤1.78 mmol/L increased from 41.3 to 61.6% during the SFOH follow-up period. These improvements in serum phosphate control were maintained for the duration of the 1-year follow-up period and achieved using slightly fewer phosphate binder pills than were prescribed at baseline (6.3 pills/day vs 5.0 to 5.3 pills/day, Q1–Q4).

A subgroup analysis that categorized patients according to their prior and concomitant phosphate binder therapy use also showed that SFOH treatment, administered either as monotherapy to phosphate binder-naïve patients (mSFOH), or as add-on therapy (PB + SFOH), improved serum phosphate control, without increasing overall phosphate binder pill burden in the latter group. No significant improvements in serum phosphate control were observed for phosphate binder-pretreated patients switched to SFOH monotherapy (PB → mSFOH). It is noteworthy that the majority of patients in this subgroup (58.5%) were already achieving serum phosphate levels ≤1.78 mmol/L on their baseline phosphate binder regimen, indicating the main clinical objective for switching them to SFOH monotherapy may have been to maintain control of their existing serum phosphate levels, rather than to achieve further phosphate reductions. Furthermore, the number of patients included in this subgroup was small (*n* = 53), making it difficult to draw any firm conclusions from the analysis.

Our findings are consistent with retrospective database studies performed on US hemodialysis patients, which have shown significant improvements in serum phosphate control following prescription of SFOH [14, 15, 17]. One US database study of 530 hemodialysis patients who had switched from another phosphate binder to SFOH monotherapy for 1 year reported a twofold increase from baseline (17.7%) in the proportion of patients achieving in-range serum phosphate (36% after 1 year), and a 50% reduction in phosphate binder pill burden (8.5 to 4.0–4.3 pills/day) [17]. It is important to highlight the major differences between the patient populations evaluated in this US database study versus our analysis, particularly with respect to the severity of hyperphosphatemia, which was greater in those patients evaluated in the US study. Furthermore, in contrast to the reduction in daily phosphate binder pill burden observed in the US study [17], our analysis showed a small increase in phosphate binder pill burden (from 2.1 to 2.6–2.8 pills/day) for the phosphate binder-pretreated patients who switched to SFOH monotherapy. However, SFOH pill burden for patients in our analysis was lower than that reported for the US hemodialysis patients (~ 2.5 pills/day vs ~ 4.0 pills/day). This is likely due to differences in disease severity and dietary habits between these patient populations.

The analysis of iron-related parameters in the overall study cohort found small increases in serum ferritin, TSAT and hemoglobin during SFOH treatment, which were consistent with the results observed in the Phase 3 study [12, 13, 25]. There were significant reductions in the mean ESA dose per week from baseline to Q1–Q4 and a progressive decrease in the proportion of patients receiving ESA during the follow-up period. Furthermore, the mean dose of IV iron therapy decreased significantly from baseline to Q2 and Q3. These findings are in line with the results observed in a post hoc analysis of the Phase 3 study, which showed a reduction in the use of IV iron and ESA therapies among patients treated with SFOH over 52 weeks [25]. It is difficult to determine whether the observed decline in IV iron and ESA therapy usage and dose in our study are specifically related to SFOH. However, data from previous clinical studies indicate that gastrointestinal iron absorption from SFOH is minimal [25, 26].

The present study had some limitations: it was retrospective and observational, and data were extracted from routine clinical care records rather than collected explicitly for research purposes. Therefore, information on treatment indication for phosphate binder therapy, treatment adherence and tolerance, adverse events and reasons for phosphate binder discontinuation were not available. Furthermore, some relevant parameters, including residual renal function were not collected, which may have led to unmeasured confounding. By design, the study analyzed only hemodialysis patients who had received treatment with SFOH introducing selection bias. Hence, the patient population analyzed may not have been fully representative of all hemodialysis patients with hyperphosphatemia in the EuCliD® cohort. Other limitations include the lack of an active control arm, as the baseline was used as the comparator. Differences in prescribing practices between participating countries introduces potential selection bias, which may account for observed differences in patient demographics – for example, the target serum phosphate levels and comorbidities. Inclusion of patients with varying durations of exposure to SFOH therapy and the loss of subjects for other reasons (e.g., kidney transplantation, death, transferred to another clinic) meant the number of subjects available for analysis progressively decreased during the course of the 1-year SFOH follow-up period, which was a further limitation of the analysis.

## Conclusions

This retrospective database analysis of > 1000 hemodialysis patients from five European countries showed that treatment with SFOH, when prescribed as monotherapy to phosphate binder-naïve patients or in addition to existing phosphate binder therapy in routine clinical practice, was associated with a significant improvement in serum phosphate control, and a relatively low daily pill burden.

## Data Availability

The datasets used and/or analysed during the current study available from the corresponding author on reasonable request.

## Abbreviations

CKD-MBD: Chronic kidney disease-mineral and bone disorder
COSMOS: Current Management of Secondary hyperparathyroidism – a Multicenter Observational Study
ESA: Erythropoiesis-stimulating agent
IV: Intravenous
KDIGO: Kidney Disease: Improving Global Outcomes
KDOQI: Kidney Disease Outcomes Quality Initiative
PB: Phosphate binder
PTH: Parathyroid hormone
SD: Standard deviation
SFOH: Sucroferric oxyhydroxide
TSAT: Transferrin saturation
